# Contribution of syndecan-4 genetic variants to hypertension, the TAMRISK study

**DOI:** 10.1186/1756-0500-7-815

**Published:** 2014-11-19

**Authors:** Tarja Kunnas, Seppo T Nikkari

**Affiliations:** Department of Medical Biochemistry, University of Tampere Medical School and Fimlab laboratories, Tampere, 33014 Finland

## Abstract

**Background:**

A human syndecan-4 genetic variant (rs1981429) has previously been associated with lean tissue mass and intra-abdominal fat, and SNP rs4599 with resting energy expenditure in healthy early pubertal children. These variations could thus cause overweight and hypothetically lead to hypertension. Their association with body mass index and blood pressure was therefore studied in a Finnish cohort of adults.

**Methods:**

The data was collected from the Tampere adult population cardiovascular risk study (TAMRISK). A total of 279 cases with hypertension and/or coronary artery disease (CAD), and 488 non-hypertensive healthy controls were selected from a Finnish periodic health examination 50-year-old cohort. Information was available also from their 45-year examination. DNA was extracted from buccal swabs and human syndecan-4 gene SNPs were analyzed using KASP genotyping.

**Results:**

The SNP rs1981429 variant TT was significantly associated with hypertension, as compared to variants TG and GG at the age of 50 years (p=0.015). The variant TT was also associated with increased BMI at the ages of 45 and 50 years (p=0.008 and p=0.026, respectively). In addition, TT genotype associated with increased CAD prevalence (P=0.013). No significant associations between rs4599 variants and hypertension or BMI were found. In haplotype analysis the number of alleles T (rs1981429)/C (rs4599) was linearly associated with CAD prevalence; the highest prevalence (13%) was in haplotype TT/CC and lowest prevalence (1%) in haplotype GG/TT (p=0.01).

**Conclusion:**

Syndecan-4 polymorphisms were associated with essential hypertension, BMI, and CAD prevalence in the TAMRISK study.

## Background

There are four syndecans in mammals [1]. Syndecans are cell surface proteoglycans with heparan sulfate chains that bind to other ECM components, growth factors, clotting factors, and even to enzymes involved in lipid metabolism [2]. Syndecan-4 is widely expressed in mesodermal tissues, including the heart and vasculature [3]. It has also been suggested that modification of LDL triggers an increased uptake by macrophages through a syndecan-4-dependent pathway leading to foam cell formation [4]. Syndecan-4 is upregulated in the hypertrophic left ventricle following myocardial infarction, suggesting a role for this proteoglycan family in cardiac remodeling [5]. In fact, experimental data has suggested that syndecan-4 plays an important role in the immune response of the heart to increased pressure, influencing cardiac remodeling [6].

Single nucleotide polymorphisms (SNPs) in the human syndecan-4 gene have previously been tested for association with body composition, metabolism, glucose homeostasis, and sleep traits in a cohort of healthy early pubertal children [7]. SNP rs4599 was significantly associated with resting energy expenditure, fasting glucose levels and sleep duration. On average, children homozygous for the minor allele C had lower levels of glucose, higher resting energy expenditure, and slept shorter than children homozygous for the common allele T. SNP rs1981429 was associated with lean tissue mass and intra-abdominal fat. These previous results suggest that syndecan family members play a key role in the regulation of body metabolism.

Lifestyle factors are the major causes of hypertension in Western countries, including overweight, physical inactivity, high salt intake and low potassium intake [8]. The genetic component in hypertension is estimated as 30 – 50% of the total impact [9]. On the other hand, although candidate-gene and genome-wide association studies (GWAS) have previously identified numerous genetic loci that are associated with blood pressure, collectively these explain only a few percent of the heritability for hypertension [10, 11].

To our knowledge, there are no previous studies that have addressed the association of syndecan-4 genetic variants with hypertension. We wanted to assess the role of syndecan-4 variants in a Finnish population, by analyzing cohorts from the Tampere adult population cardiovascular risk study (TAMRISK) [12].

## Methods

### Subjects

The data for the TAMRISK study was collected from periodic health examinations (PHE) done for 50-year-old men and women living in Tampere, Finland [12]. TAMRISK data includes information of risk factors for hypertension: blood pressure, weight, family history of cardiovascular diseases, lipid values and smoking, diabetes and exercise habits. Buccal swabs for DNA extraction and a permissions form to use PHE data were collected by mail subsequent to the physical examination. The DNA samples were collected during years 2006–2010. Informed consent was obtained from all participants. The Ethics Committees of the Tampere University Hospital and the City of Tampere approved the study.

Cases (n=279) in this study were the subjects who had hypertension and/or CAD at the age of 50 years (as diagnosed by a physician) and for each case, at least one normotensive control (n=448) with the same sex and similar smoking habits, were chosen from a PHE cohort (n=6000). Smoking status was evaluated based on self-reporting. Finally, we selected a subpopulation of men and women who had available data of blood pressure measurements at the age of 45- and 50 years.

### Baseline measurements

The basic evaluation in 1988–91 included an interview by a public health nurse. The interview was conducted using a structured questionnaire about health and health-related behaviour, including questions about current and previous diseases. Information on current and previous diseases was based on self-report of diagnosis by a physician, including history of coronary artery disease, myocardial infarction and diabetes. Family history of hypertension in a close relative was also asked in the questionnaire. The frequency of physical exercise comprised both leisure and commute related activity. Physical examination included a single blood pressure (BP) measurement (mm of mercury) using a calibrated mercury sphygmomanometer. Height (cm) and weight (kg) were recorded from which the body mass index was calculated.

### Genotyping

DNA was extracted from buccal swabs using a commercial kit (Qiagen Inc., Valencia, Calif., USA). The samples were transferred into 96-well plates and genotyped for SDC4 SNPs rs4599 and rs1981429 at LGC Genomics (Herz, UK) using the Competitive Allele Specific PCR (KASP) technique.

### Statistical analysis

Associations of the two genotyped SNPs with risk factors were analyzed using dominant, additive, and recessive models. We used dummy variables to code the three genotypes in each model [7]. In the additive model, we used 0, 1 and 2 to code for individuals homozygous for the major allele, heterozygous, and homozygous for the minor allele, respectively. In the dominant model for rs4599, genotypes T/T, T/C, C/C were coded as 0, 1, 1, respectively; in the recessive model, genotypes T/T, T/C, C/C were coded as 0, 0, 1, respectively. In the dominant model for rs1981429, genotypes T/T, T/G, G/G were coded as 0, 1, 1, respectively; in the recessive model, genotypes T/T, T/G, G/G were coded as 0, 0, 1, respectively.

Analysis of variance, *t*-test, and Chi-square test for categorical variables were applied for the comparison of genotypes. To find the interpretative factors for hypertension/CAD, we used logistic regression analysis. If the distribution was skewed, the analysis was performed using transformed values to approximately normalize the distribution. P values less than 0.05 were considered significant. Analyses were carried out using SPSS 16.0 for Windows (SPSS Inc., Chicago, Illinois, USA).

## Results

Clinical characteristics of the cases (279) and controls (488) at the age of 50 years are presented in Table 1. The case group of subjects with hypertension and/or CAD was compared to healthy controls. Cases had higher body mass index (BMI) (p<0.001), fasting glucose (p<0.001), systolic blood pressure (p<0.001) and diastolic blood pressure (p<0.001) compared to controls. In the whole study population the frequencies of the rs1981429 variants were 0.240 for GG (n=190), 0.514 for TG (n=399) and 0.245 (n=190) for TT. The frequencies of the rs1981429 variants were significantly different between cases and controls. In the whole study population the frequencies of the rs4599 variants were 0.038 for CC (n=32), 0.369 for TC (n=285) and 0.593 (n=462) for TT. These frequencies did not differ between cases and controls (Table 1).

**Table 1:** **Clinical characteristics of study population at the age of 50 years**

	Cases (n = 279)	Controls (n = 488)	P
Age (years)	50 ± 0	50 ± 0	
Gender (male) %	64	62	0.699
Body mass index (kg/m2)	28.8 ± 5.2	25.9 ± 4.0	<0.000
Cholesterol (mmol/l)	5.41 ± 1.08	5.39 ± 0.88	0.769
Glucose (mmol/l)	5.24 ± 1.40	4.85 ± 0.52	<0.000
Systolic blood pressure (mm Hg)	142.4 ± 16.4	131.3 ± 15.9	<0.000
Diastolic blood pressure (mm Hg)	92.3 ± 8.6	85.8 ± 9.8	<0.000
Exercise (at least twice a week) %	36.0	30.0	0.090
Diabetes %	8.0	0	<0.000
Myocardial infarction %	6.0	0	<0.000
Coronary artery disease %	11.0	0	<0.000
Syndecan-4 rs4599 (CC/TC/TT) %	4.4/36.1/59.5	3.3/37.6/59.1	0.717
Syndecan-4 rs1981429 (GG/TG/TT) %	22.4/48.0/29.6	25.4/53.3/21.3	0.017

Values are means ± SD.

For further analyses, the case and control groups were combined (Table 2). Significant associations with hypertension were found in the dominant model, comparing rs1981429 variant TT to the G allele carriers at the age of 50 years (p=0.015). Individuals homozygous for the T allele had 0.8 mmHg and 2.3 mmHg higher diastolic blood pressure at the age of 45 compared to genotypes GG and TG, respectively (p=0.025). At the age of 45 years, 9% of the combined groups were on blood pressure medication, compared to 30% at the age of 50 years. The variant TT was associated with increased BMI at the ages of 45 and 50 years (p=0.008 and p=0.026, respectively). Individuals homozygous for the T allele had 2% and 3% higher BMI at the ages of 45 and 50 years than those homozygous for the G allele, respectively. All of the above findings and significant differences were also observed when analyzing the data in the additive model (Table 2).

**Table 2:** **Means (SD) for hypertension, coronary artery disease, blood pressure and body mass index according to syndecan-4 polymorphisms**

				P*		
**rs4599**	**C/C**	**T/C**	**T/T**	**Additive**	**Dominant (TT)**	**Recessive (CC)**
**n**	32	287	448			
**Hypertension %**	39	31	35	0.496	0.225	0.334
**Coronary artery disease % (n=31)**	10	5	3	0.057	0.061	0.108
**Systolic blood pressure (mm Hg) at 45**	133.6 (15.3)	132.2 (13.5)	133.0 (14.3)	0.735	0.542	0.750
**Systolic blood pressure (mm Hg) at 50**	136.8 (12.7)	134.7 (16.7)	135.3 (17.1)	0.771	0.738	0.485
**Diastolic blood pressure (mm Hg) at 45**	83.6 (7.5)	85.1 (9.0)	85.6 (9.5)	0.471	0.329	0.330
**Diastolic blood pressure (mm Hg) at 50**	87.3 (8.4)	88.0 (10.1)	88.1 (9.7)	0.904	0.780	0.640
**Body mass index (kg/m** ^**2**^ **) at 45**	27.4 (4.5)	26.5 (4.3)	26.3 (4.2)	0.368	0.368	0.215
**Body mass index (kg/m** ^**2**^ **) at 50**	27.6 (4.5)	26.9 (4.7)	26.9 (4.7)	0.776	0.776	0.438
**rs1981429**	**G/G**	**T/G**	**T/T**	**Additive**	**Dominant (TT)**	**Recessive (GG)**
**n**	185	396	186			
**Hypertension %**	32	32	41	0.074	**0.015**	0.283
**Coronary artery disease % (n=31)**	2	3	7	**0.013**	**0.010**	0.056
**Systolic blood pressure (mm Hg) at 45**	133.6 (15.7)	131.5 (13.7)	134.2 (14.2)	0.086	0.108	0.379
**Systolic blood pressure (mm Hg) at 50**	133.1 (16.7)	135.4 (17.3)	136.2 (15.9)	0.157	0.252	0.064
**Diastolic blood pressure (mm Hg) at 45**	85.9 (10.0)	84.4 (8.8)	86.7 (9.4)	**0.018**	**0.025**	0.408
**Diastolic blood pressure (mm Hg) at 50**	87.1 (9.6)	88.4 (10.1)	87.9 (9.2)	0.330	0.928	0.161
**Body mass index (kg/m** ^**2**^ **) at 45**	26.4 (4.0)	26.1 (4.0)	27.2 (4.7)	**0.024**	**0.008**	0.832
**Body mass index (kg/m** ^**2**^ **) at 50**	27.1 (4.5)	26.6 (4.4)	27.6 (5.2)	**0.038**	**0.026**	0.629

*indicate P values calculated assuming additive, dominant, and recessive models. P values <0.05 are highlighted in bold case.

The rs1981429 variant TT was also associated with increased CAD prevalence (P=0.010). Individuals homozygous for the T allele had 71% more CAD than those homozygous for the G allele. In logistic regression the rs1981429 variant TT carriers had a 2.7 higher risk (p=0.009, CI 1.3 – 5.4) of CAD compared to the G allele carriers. The results remained the same (2.5 fold risk) after adjusting with BMI and gender.

**Figure 1:**
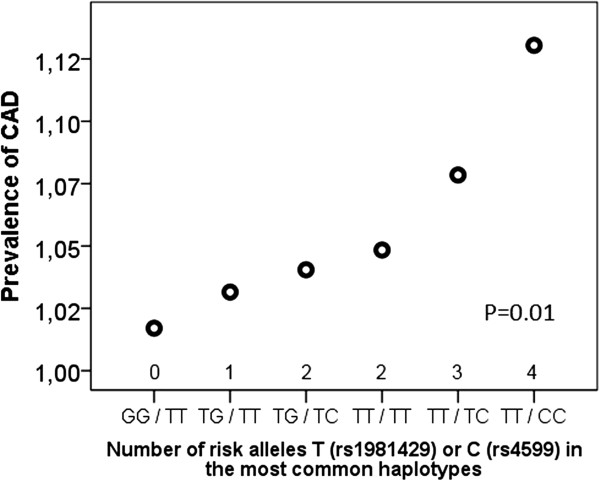
**Association of the most common syndecan-4 haplotypes with coronary artery disease (CAD) prevalence.**

No significant association of the rs4599 variant with hypertension, blood pressure or BMI was found. However, there was a nonsignificant trend for association of the C-allele with CAD. The haplotype frequencies for SNPs rs1981429/rs4599 are shown in Table 3. In haplotype analysis of the most common haplotypes (with more than 20 subjects each), the number of alleles T (rs1981429)/C (rs 4599) was linearly associated with CAD prevalence; the highest prevalence (13%) was in haplotype TT/CC and lowest prevalence (1%) in haplotype GG/TT (p=0.01) (Figure 1). In logistic regression the risk for CAD was 8.6 fold higher (p=0.01, CI 1.6 – 45.7) in haplotype TT/CC compared to GG/TT.

**Table 3:** **All haplotypes for SNPs rs1981429/rs4599**

	Frequency	Percent
GG/CC	1	0.1
TG/CC	5	0.7
TT/CC	23	3.0
GG/TC	11	1.4
TG/TC	173	22.6
TT/TC	101	13.2
GG/TT	171	22.3
TG/TT	220	28.7
TT/TT	62	8.1
**Total**	767	100.0

## Discussion

We report that syndecan-4 gene SNP rs1981429 variant TT was significantly associated with hypertension, as compared to variants TG and GG at the age of 50 years. To our knowledge, there are no previous studies showing such an association. There was also an association of this genotype with diastolic blood pressure at the age of 45 years. The observed effect of a single gene variation on blood pressure in GWAS studies is low: +1.16 mmHg at the highest [10], as also seen in the present study. Individuals homozygous for the T allele had 0.8 mmHg and 2.3 mmHg higher diastolic blood pressure at the age of 45 compared to genotypes GG and TG, respectively. A possible difference in blood pressure at the age of 50 might be difficult to establish, since practically all of the subjects who had hypertension were on blood pressure medication at this age.

There were only 31 subjects with CAD, but SNP rs1981429 variant TT was associated with increased CAD prevalence. Furthermore, in haplotype analysis the number of alleles T (rs1981429)/C (rs 4599) was linearly associated with CAD prevalence; the highest prevalence was in haplotype TT/CC and lowest prevalence in GG/TT.

Our findings of the association between syndecan-4 SNP rs1981429 with hypertension and CAD are interesting, because serum syndecan-4 concentration has shown promise as a novel diagnostic and prognostic biomarker for heart failure. Serum syndecan-4 has been suggested to represent a biomarker of pathological left ventricle remodeling [5, 13–15]. Syndecan-4 levels are increased in the hypertrophic myocardium of patients with aortic stenosis [14], and increased syndecan-4 levels have also been demonstrated in the post-infarcted heart [5, 16]. In cardiac cells, syndecan-4 is localized to cellular adhesion sites which are important for signaling across the membrane [1, 17].

The variant TT of rs1981429 was also associated with increased BMI at the ages of 45 and 50 years. This is in line with previous findings by De Luca et al. that SNPs in the human syndecan-4 gene were associated with body composition in a cohort of healthy early pubertal children [7]. However in the previous study, the variant GG of SNP rs1981429 was associated with increased intra-abdominal fat and less lean mass, while we report an association of the variant TT with BMI. On the other hand, the previous study population was smaller (n=252), and represented a mixture of healthy early pubertal children with African American, Caucasian and Hispanic ancestry, while our study population (n=767) represented Caucasian adults. Nevertheless, these previous and our present results suggest that syndecan family members may play a role in the regulation of body metabolism.

Syndecan-4 is essential in skeletal muscle development and regeneration [18] and plays a role in the regulation of focal adhesion kinase phosphorylation [19], which in turn mediates the insulin sensitivity of skeletal muscle cells [20]. Consequently, De Luca et al. have suggested that it is plausible that syndecan-4 is involved in energy regulation via an insulin-signaling mechanism that impacts overall body metabolism [7]. The mechanisms behind associations of syndecan-4 polymorphisms with essential hypertension, BMI, and CAD need further studies.

## Conclusions

In conclusion, syndecan-4 polymorphisms were associated with essential hypertension, BMI, and CAD prevalence in the TAMRISK study.

## References

[1] Alexopoulou AN, Multhaupt HA, Couchman JR (2007). Syndecans in wound healing, inflammation and vascular biology. Int J Biochem Cell Biol.

[2] Esko JD, Selleck SB (2002). Order out of chaos: assembly of ligand binding sites in heparan sulfate. Annu Rev Biochem.

[3] Samarel AM (2013). Syndecan-4: a component of the mechanosensory apparatus of cardiac fibroblasts. J Mol Cell Cardiol.

[4] Boyanovsky BB, Shridas P, Simons M, van der Westhuyzen DR, Webb NR (2009). Syndecan-4 mediates macrophage uptake of group V secretory phospholipase A2-modified LDL. J Lipid Res.

[5] Finsen AV, Woldbaek PR, Li J, Wu J, Lyberg T, Tonnessen T, Christensen G (2004). Increased syndecan expression following myocardial infarction indicates a role in cardiac remodeling. Physiol Genom.

[6] Strand ME, Herum KM, Rana ZA, Skrbic B, Askevold ET, Dahl CP, Vistnes M, Hasic A, Kvaløy H, Sjaastad I, Carlson CR, Tønnessen T, Gullestad L, Christensen G, Lunde IG (2013). Innate immune signaling induces expression and shedding of the heparan sulfate proteoglycan syndecan-4 in cardiac fibroblasts and myocytes, affecting inflammation in the pressure-overloaded heart. FEBS J.

[7] De Luca M, Klimentidis YC, Casazza K, Chambers MM, Cho R, Harbison ST, Jumbo-Lucioni P, Zhang S, Leips J, Fernandez JR (2010). A conserved role for syndecan family members in the regulation of whole-body energy metabolism. PLoS One.

[8] Geleijnse JM, Kok FJ, Grobbee DE (2004). Impact of dietary and lifestyle factors on the prevalence of hypertension in Western populations. Eur J Public Health.

[9] Kupper N, Willemsen G, Riese H, Posthuma D, Boomsma DI, de Geus EJC (2005). Heritability of daytime ambulatory blood pressure in an extended twin design. Hypertension.

[10] Levy D, Ehret GB, Rice K, Verwoert GC, Launer LJ, Dehghan A, Glazer NL, Morrison AC, Johnson AD, Aspelund T, Aulchenko Y, Lumley T, Köttgen A, Vasan RS, Rivadeneira F, Eiriksdottir G, Guo X, Arking DE, Mitchell GF, Mattace-Raso FU, Smith AV, Taylor K, Scharpf RB, Hwang SJ, Sijbrands EJ, Bis J, Harris TB, Ganesh SK, O’Donnell CJ, Hofman A (2009). Genome-wide association study of blood pressure and hypertension. Nat Genet.

[11] Munroe PB, Johnson T, Caulfield M (2009). The genetic architecture of blood pressure variation. Curr Cardiovasc Risk Rep.

[12] Kunnas T, Määttä K, Palmroos P, Nikkari ST (2012). Periodic cohort health examinations in the TAMRISK study show untoward increases in body mass index and blood pressure during 15 years of follow-up. BMC Public Health.

[13] Takahashi R, Negishi K, Watanabe A, Arai M, Naganuma F, Ohyama Y, Kurabayashi M (2011). Serum syndecan-4 is a novel biomarker for patients with chronic heart failure. J Cardiol.

[14] Finsen AV, Lunde IG, Sjaastad I, Østli EK, Lyngra M, Jarstadmarken HO, Hasic A, Nygard S, Wilcox-Adelman SA, Goetinck PF, Lyberg T, Skrbic B, Florholmen G, Tønnessen T, Louch WE, Djurovic S, Carlson CR, Christensen G (2011). Syndecan-4 is essential for development of concentric myocardial hypertrophy via stretch-induced activation of the calcineurin–NFAT pathway. PLoS One.

[15] Herum KM, Lunde IG, Skrbic B, Florholmen G, Behmen D, Sjaastad I, Carlson CR, Gomez MF, Christensen G (2013). Syndecan-4 signaling via NFAT regulates extracellular matrix production and cardiac myofibroblast differentiation in response to mechanical stress. J Mol Cell Cardiol.

[16] Matsui Y, Ikesue M, Danzaki K, Morimoto J, Sato M, Tanaka S, Kojima T, Tsutsui H, Uede T (2011). Syndecan-4 prevents cardiac rupture and dysfunction after myocardial infarction. Circ Res.

[17] VanWinkle WB, Snuggs MB, De Hostos EL, Buja LM, Woods A, Couchman JR (2002). Localization of the transmembrane proteoglycan syndecan-4 and its regulatory kinases in costameres of rat cardiomyocytes: a deconvolution microscopic study. Anat Rec.

[18] Cornelison DD, Wilcox-Adelman SA, Goetinck PF, Rauvala H, Rapraeger AC, Olwin BB (2004). Essential and separable roles for Syndecan-3 and Syndecan-4 in skeletal muscle development and regeneration. Genes Dev.

[19] Wilcox-Adelman SA, Denhez F, Goetinck PF (2002). Syndecan-4 modulates focal adhesion kinase phosphorylation. J Biol Chem.

[20] Bisht B, Goel HL, Dey CS (2007). Focal adhesion kinase regulates insulin resistance in skeletal muscle. Diabetologia.

